# Gestational age limits for abortion and cross‐border reproductive care in Europe: a mixed‐methods study

**DOI:** 10.1111/1471-0528.16534

**Published:** 2020-10-23

**Authors:** S De Zordo, G Zanini, J Mishtal, C Garnsey, A‐K Ziegler, C Gerdts

**Affiliations:** ^1^ Department of Anthropology University of Barcelona Barcelona Spain; ^2^ Department of People and Organisations School of Business and Management Queen Mary, University of London London UK; ^3^ Department of Anthropology University of Central Florida Orlando FL USA; ^4^ Ibis Reproductive Health Cambridge MA USA; ^5^ University College Dublin Dublin Republic of Ireland; ^6^ Ibis Reproductive Health Oakland CA USA

**Keywords:** Abortion, delays, gestational age limits, reproductive health, travel

## Abstract

**Objectives:**

Little is known about the experiences of women who travel within Europe for abortion care from countries with relatively liberal laws. This paper aims to assess the primary reasons for travel among a sample of women who travelled from European countries with relatively liberal abortion laws to obtain abortion care mainly in the UK and the Netherlands.

**Design:**

Multi‐country, 5‐year mixed methods study on barriers to legal abortion and travel for abortion.

**Setting:**

UK, the Netherlands and Spain.

**Population or Sample:**

We present quantitative data from 204 surveys, and qualitative data from 30 in‐depth interviews with pregnant people who travelled to the UK, the Netherlands and Spain from countries where abortion is legal on broad grounds within specific gestational age (GA) limits.

**Methods:**

Mixed‐methods.

**Main outcome measures:**

GA when presenting at abortion clinic, primary reason for abortion‐related travel.

**Results:**

Study participants overwhelmingly reported travelling for abortion because they had exceeded GA limits in their country of residence. Participants also reported numerous delays and barriers to receiving care.

**Conclusions:**

Our findings highlight the need for policies that support access to abortion throughout pregnancy and illustrate that early access to it is necessary but not sufficient to meet people’s reproductive health needs.

**Funding:**

This study is funded by the European Research Council (ERC).

**Tweetable abstract:**

This study shows that GA limits drive women from EU countries where abortion is legal to seek abortions abroad.

## Tweetable abstract

This study shows that GA limits drive women from EU countries where abortion is legal to seek abortions abroad.

## Introduction

Abortion is legal upon request, or on broad social or economic grounds, in nearly all European countries.^1^ However, gestational age limits, waiting periods, conscientious objection, as well as other regulatory and procedural barriers vary in their existence and application in countries across the continent.^2^, ^3^ Such laws and regulations create a patchwork legal landscape, and limit access to abortion.^4^, ^5^, ^6^ A recent 158‐country analysis of abortion policies demonstrates that barriers to abortion access, including regulatory requirements, may delay abortion care‐seeking – causing some people seeking abortion to exceed the gestational limits specified by a country’s abortion laws.^7^

Recent evidence suggests that people seeking abortion care in countries where abortion is legal, but where legal and procedural barriers exist, may travel abroad to obtain abortion services. According to publicly available data, in 2017, 3495 non‐Dutch resident women sought abortion care in the Netherlands, the majority of whom travelled from Germany, France and Belgium.^8^ Similarly, in 2018, 3132 non‐British resident women travelled to England and Wales to receive abortion services; approximately 95% of those women did so from the Republic of Ireland prior to the 2018 modification of the abortion law and the rest travelled from Italy, France, Germany and Denmark.^9^

Although abortion travel is a relatively well‐documented phenomena, the majority of studies focus on the experiences of people travelling within the USA^10^ or from European countries with restrictive laws, such as Ireland.^11^, ^12^, ^13^ The existing literature on abortion‐related travel has shown that a lack of trained and willing abortion providers, restrictions on insurance coverage, gestational age limits, parental or spousal consent laws, and other legal constraints contribute to the need to travel considerable distances, sometimes even crossing borders, to access abortion care.^7^, ^10^, ^14^, ^15^, ^16^, ^17^, ^18^, ^19^, ^20^ Travel for abortion care represents a considerable burden for people seeking this service, who cite the cost of travel, in addition to the logistical (e.g. child care) and emotional components of the experience, as difficult and disruptive to their lives.^21^, ^22^, ^23^, ^24^, ^25^, ^26^

Little is known about the reasons behind travelling, or the experiences and burdens faced by those who travel within Europe for abortion from countries with relatively liberal laws.^22^, ^27^ This paper aims to assess the primary reasons for travel among a sample of pregnant people who travelled from countries with relatively liberal abortion laws to obtain abortion care mainly in the UK and the Netherlands.

## Methods

Data for this analysis are drawn from a multi‐country, 5‐year, mixed‐methods study on barriers to legal abortion and travel for abortion, funded by the European Research Council (ERC). The study aims to assess the impact of legal, procedural and social barriers to abortion care, and to document and explore the experiences of pregnant people who travel abroad to seek abortion services in the UK, Netherlands and Spain or who travel domestically within their country of residence in France, Italy and Spain.

In this paper, we present data from study participants who travelled abroad from countries where abortion is legally permitted on request, or on broad social or economic grounds within specified gestational age (GA) limits. Data presented here were collected via questionnaires and in‐depth interviews (IDIs) between July 2017 and March 2019 at three British Pregnancy Advisory Service (BPAS) clinics in the UK and two abortion clinics in the Netherlands, and via IDIs collected between March 2018 and April 2019 at three abortion clinics in Spain. Participating clinics were selected based on available data regarding the number of non‐residents who obtained abortion care at the respective clinics in the years preceding the launch of the study.

Pregnant people eligible to participate in this study were 18 years of age or older, had travelled from another European Union country to seek abortion care, and were proficient in French, Italian, English, German or Spanish. Suitable individuals were identified by an on‐site researcher and/or clinic staff, and approached in a sequential 2:1 ratio (travelling from country with relatively liberal abortion law: travelling from country with restrictive abortion law) and were then provided with a study information sheet upon their arrival to the clinic. Those who expressed interest in participating could complete an anonymous, self‐administered, tablet‐based questionnaire, and/or take part in a confidential in‐depth interview while they waited for their medical consultation, or at any time prior to the abortion procedure.

Questionnaire responses took place at the clinics, and IDIs were carried out at the clinics or remotely by phone or via internet. Electronic consent was collected for all questionnaire participants, and verbal and written consent was collected for all interview participants. Questionnaire participants received €10 or a €10 gift card, whereas interview participants received a €25 gift card as compensation for their time.

Participants who completed the questionnaire answered questions about their socio‐demographic characteristics, reproductive history, index pregnancy, abortion‐seeking experiences (both in their country of origin and abroad), experiences travelling abroad for abortion (time and cost), abortion stigma and experience with self‐administered abortion.

In‐depth interviews allowed us to deepen our understanding of participants’ experiences with the same range of topics covered in the questionnaire, and focused primarily on experiences with barriers to abortion care in their country of origin and abortion‐related travel abroad.

All study participants were assigned identification numbers, and IDI participants are referred to by means of pseudonyms. Quantitative analyses were conducted using STATA statistical software package: Release 12.^28^ Simple counts and percentages were calculated for most variables. IDIs were coded using Atlas.TI and analysed following a grounded theory approach.^29^ SDZ, GZ, and AKZ conducted the interviews and coded them. They used both pre‐determined codes that were initially created based on the main IDI guide themes, and new codes that emerged from the interviews. For instance, while the topic of ‘delays‘ with regard to access to care was a predetermined topic of inquiry, other emergent explanations were also identified (e.g. misinformation regarding gestational age from healthcare providers). All new codes were periodically discussed, adopted by the entire team, and used to re‐code all interviews. Discrepancies and disagreements were discussed during the coding process as well as throughout the writing process. No significant disagreements in the interpretation of the data emerged. For this article, we focused particularly on the code groups: reasons for travelling, barriers, abortion information seeking and delays.

For the purposes of this analysis, countries were characterised as having relatively liberal abortion laws if, during the period of data collection, abortion was available upon request or on broad grounds within legally specified GA limits. Our primary outcomes were self‐reported measures of GA upon arrival at an abortion clinic and primary reason for abortion‐related travel.

## Results

### Quantitative findings

Of all eligible participants approached between July 2017 and March 2019, 43.7% consented to participate in the quantitative questionnaire. In total, 204 pregnant people who travelled from countries with relatively liberal abortion laws participated in our questionnaire; the majority of them (88%) were recruited in the Netherlands. Table 1 presents the socio‐demographic characteristics of questionnaire participants. Participants travelled from eight European countries (Austria, Belgium, Bulgaria, Denmark, France, Germany, Italy and Luxemburg) with the majority (56%) travelling from Germany, followed by 23% proceeding from France. Most participants indicated that they had sufficient economic resources to meet their basic needs all (39%) or most (27%) of the time, although 18% reported moderate to severe financial insecurity. Most participants had not had a prior abortion (76%) and had not given birth (61%).

**Table 1:** Socio‐demographic profile of participants (*n* = 204*)

	% (*n*)
Country of residence	
Germany	56 (115)
France	23 (47)
Italy	8 (16)
Belgium	6 (13)
Other (Austria (7), Denmark (4), Luxemburg (1), Bulgaria (1))	7 (13)
Age	
18–24	44 (90)
25–34	42 (86)
35+	14 (28)
Number of children	
0	61 (125)
1–2	30 (62)
3+	8 (17)
Prior abortion	
Yes	23 (46)
Missing/prefer not to answer	1 (2)
Marital status	
Married or in a civil partnership/cohabitating	43 (87)
Single, separated, or divorced	42 (86)
Other	2 (5)
Missing/prefer not to answer	13 (26)
Highest level of education completed	
Secondary school or below	34 (69)
Some university	20 (40)
University	23 (47)
Postgraduate	10 (21)
Missing/Prefer not to answer	13 (27)
Employment (participants could select more than one option)	
Employed (full‐time or part‐time)	57 (102)
Unemployed	10 (17)
Student	25 (45)
Other (unable to work, homemaker, self‐employed/Freelancer)	13 (26)
Missing/prefer not to answer	13 (26)
Sufficient income for basic needs	
All of the time	39 (80)
Most of the time	27 (55)
Some of the time	7 (15)
Rarely	8 (17)
Never	3 (7)
Don’t know/missing/prefer not to answer	15 (29)
Religious affiliation	
Catholic	32 (66)
Protestant	14 (29)
Islam	6 (12)
Atheist or doesn’t identify with a religion	25 (52)
Other	5 (9)
Missing/prefer not to answer	18 (36)

* Nine collected surveys were deemed ineligible and excluded from analysis. Responses were deemed ineligible when participants who did not meet all requirements were mistakenly recruited (5), participants decided not to go through with their scheduled abortion procedure (2), participants only filled out the first few questions of the survey (2).

Table 2 presents data related to the timing of participants’ pregnancies and seeking abortion. Participants reported confirming their pregnancy at a median gestational age of 12^+0^ weeks (IQR: 10^+0^ –14^+0^ weeks). A minority (15%) of participants confirmed their pregnancy prior to 6 weeks and 56% confirmed their pregnancy at 14 weeks of gestation or later. A total of 33% of participants considered abortion before 14^+0^ weeks (median: 14^+6^ weeks; IQR: 13^+0^–17^+0^ weeks). Participants showed up for abortion care at a median 18 weeks of gestation (IQR: 16^+0^–20^+0^ weeks).

**Table 2:** Participants’ gestational ages* at the time of first confirming pregnancy, considering abortion, and presenting for care at clinics in the UK and the Netherlands

	% (*n*)
Gestational age at time of confirming pregnancy (*n* = 204, median: 12^+0^ weeks; IQR: 10^+0^ –14^+0^ weeks)	
≤6^+6^ weeks	15 (31)
7^+0^ to 13^+6^ weeks	23 (46)
14^+0^ to 19^+6^ weeks	50 (101)
≥20^+0^ weeks	6 (13)
Don’t know/prefer not to answer	6 (13)
Gestational age at time of first considering abortion (*n* = 204, median: 14^+6^ weeks; IQR: 13^+0^ to 17^+0^ weeks)	
≤6^+6^ weeks	10 (20)
7^+0^ to 13^+6^ weeks	23 (47)
14^+0^ to 19^+6^ weeks	50 (101)
≥20^+0^ weeks	8 (17)
Don’t know/prefer not to answer	9 (19)
Gestational age when presenting for care at abortion clinic where recruited (*n* = 204, median: 18^+0^ weeks; IQR: 16^+0^ to 20^+0^ weeks)	
≤13^+6^ weeks	6 (12)
14^+0^ to 19^+6^ weeks	74 (151)
≥20^+0^ weeks	19 (39)
Missing/prefer not to answer	1 (2)

* Participants self‐evaluated their gestational age while filling out the survey.

Table 3 describes self‐reported reasons motivating participants’ cross‐border travel for abortion care. In all, 79% of participants cited gestational age limits, i.e. that it was too late for them to have an abortion in their home country, as the primary reason for travelling in search of abortion care in the UK or the Netherlands.

**Table 3:** Participants’ self‐reported reasons for cross‐border abortion care

Primary reason for travel	% (*n*)
Abortion is not legal in my country for my situation	8 (17)
It is too late for me to have an abortion in my country	79 (162)
I was worried about a health provider refusing to help me	1 (3)
It is difficult to find a physician in my country who is willing to provide abortion care	1 (3)
I was worried about someone finding out about my abortion	2 (4)
I could not obtain an abortion for the diagnosed fetal malformation	1 (3)
I didn’t know where to get an abortion in my country	1 (2)
There are no abortion services near where I live	0
I wanted to have a surgical termination, which is not available in my country	1 (3)
I was concerned about the safety of abortion in my country	1 (1)
Other (please specify)	1 (2)
Prefer not to answer/missing	2 (4)

### Qualitative findings

We conducted interviews with 30 pregnant people: 13 from France, 11 from Italy, five from Germany and one from Austria. Our qualitative analysis complements the quantitative data presented above, and illustrates the complex range of reasons why participants in our study travelled abroad to seek abortion. These are, in order of relevance: exceeding gestational age limits, identifying the pregnancy close or beyond local legal limits, delays related to care, lack of access to abortion care, lack of information regarding local abortion services. In this section, we explore three main themes that emerged from our analysis of the interviewees’ responses in relation to their search for abortion care in their country of origin, and their reasons for travelling. We then provide representative quotes to illustrate these explanations. Our analysis expands our understanding of people’s stressful experiences when surpassing gestational age limits and embarking on abortion‐related travel.

#### Theme 1: Exceeding gestational limits: a shocking experience

Eighteen of our interviewees (ten from France, four from Germany, three from Italy and one from Austria) had already exceeded gestational age limits to undergo an abortion locally when they learned they were pregnant, confirming the questionnaire data, and had to travel abroad to seek care. When asked why they had surpassed the legal gestational age limits, our interviewees gave several reasons, including irregular periods, sometimes combined with distressful life circumstances; lack of clear pregnancy signs; misinformation given by or misunderstandings with health professionals about contraception, menstruation and/or pregnancy signs in their particular health condition.

Most of the participants in this group were shocked when they found out about their pregnancy, as they did not expect it. They all reported feeling even more astounded to find out they had exceeded GA limits for abortion care in their own country. The following cases are paradigmatic of the intense emotional distress pregnant people experience when discovering they have exceeded local GA limits to obtain an abortion.

Floryne, a French student in her late teens, was 19 weeks pregnant when she confirmed her pregnancy. She was immediately told by hospital staff that she had exceeded the local GA limits for an abortion:

Well, I was not expecting, I did not want… I wanted to do it [the abortion] in France but… In the end, [it turns out] I had exceeded the time limit… Well, I did not know right away, I had no symptoms, and I was not aware of it [the pregnancy] at all. (…) They checked my belly, and in the end, she [the provider] said: ‘Listen, madam, you’ve exceeded [the GA limit], now we have to find another solution, but I don’t know what to tell you’. So, I was left with that. We felt absolutely lost, because I didn’t expect that I had exceeded the time limit so much.

She was then referred to the Planning Familial, which provided her with information about abortion care abroad, and she had a termination in the Netherlands at 21 weeks of gestation.

Julia, a university student in her early 20s living in Germany, visited her gynaecologist because she experienced acute ovary pain and had missed her last period, but, according to her, she did not expect the pregnancy, as she suffered from hormonal problems and was prone to irregular periods. The consultation, however, revealed that she was 21 weeks pregnant and her doctor asserted that at this GA: ‘It has to be born. There is no other solution, and it has to, it definitely has to, there are no ifs and buts’. This was a serious blow for Julia, who recalls:

I thought to myself: ‘oh God, what are you doing’, and the only thing I thought for a moment was, ‘okay, I’m gonna hide in bed for a day’(…). I didn’t want to talk to anyone and just wanted some time for myself before searching for opportunities.

At home, Julia looked for information online and found a clinic in the Netherlands providing abortion care up to 22 weeks of GA, where she finally terminated her pregnancy 1 week later.

#### Theme 2: Delay in obtaining abortion care

Five other study participants, three from Italy, one from France and one from Germany, learnt they were pregnant within the GA limits for abortion in their country; however, they were delayed in their search for a provider by a number of barriers, including a lack of easily available information about abortion care or a scarcity of accessible abortion providers in their area of residence. For instance, Sveva, an Italian woman in her late 40s and mother of one, separated from her partner, explained:

I was very late [between 11 and 12 weeks] but doctors gave me the run‐around, so those 3 weeks passed, which led me to get over the 12th week, and therefore I had no alternative (…) I had a false menstruation, so I discovered that a false menstruation can exist, I am 47 years old, so I thought (…) it was due to some sort of pre‐menopause (…). [Then] I went to my usual gynaecologist, who immediately had doubts (…). I tried to tell her: ‘Look, I can’t carry on with this pregnancy, tell me what to do’, but she didn’t really listen to me (…). I left [the consultation] rather annoyed and started to ask what I could or couldn’t do in this case, they [health professionals] told me to go to a family planning centre, so I went there, and met a fantastic midwife (…). However, from the time I went there to the time I was able to find a non‐objecting doctor who could see me and listen to me, (…) another 3 weeks passed, practically.

Sveva ended up travelling to the Netherlands, where she received treatment at 17 weeks of pregnancy. Three other Italian residents participating in the study also experienced difficulties, delays and refusals when searching for abortion care at below the GA limit in their area of residence; therefore, they had to travel abroad to seek care.

Participants in our study who live in Italy and Germany described access to abortion care information as more problematic than did those living in France. In particular, refusal to furnish abortion information or care, or lack of referral and support by health professionals was discussed more frequently by our interviewees from Italy. These findings illustrate that health professionals’ refusal to give out information, let alone provide care without referral, can delay people’s access to abortion, consistently leading them to exceed local GA limits and leaving international travel as their only option to obtain abortion treatment. This situation is especially problematic in Italy, where the proportion of OBGYNs (the only health professionals certified to provide abortions) who claim conscientious objection and refuse to provide this service is extremely high (~ 70% of OBGYNs nationwide). Conscientious objection has been shown to hamper abortion access in some regions with a particular impact felt among women experiencing some forms of economic disadvantage.^3^, ^30^

#### Theme 3: Misinformation about gestational age given by healthcare providers

Three study participants (two from France, one from Italy) reported that their gestational age had been miscalculated by health professionals. This led them to think that they had more time to consider their options regarding their ongoing pregnancy and to believe they could obtain an abortion in their country of residence if they chose to. In each of these cases, the resulting miscalculation was especially problematic insomuch as it made the experience of those learning that they had exceeded GA limits for abortion even more stressful.

Among these is the case of Carla, an Italian single mother of one in her late 20s and a homemaker, which demonstrates how miscalculation of gestational age affects people’s abortion‐seeking experience, especially when combined with local GA limits. She says:

Well, originally, I wanted to carry on with the pregnancy, but then… My partner, so‐to‐speak, let’s just say that we no longer got along. And I thought I was still within the limits. But in Italy two gynaecologists got the GA wrong… They told me I was 9 weeks in but, in the end, I was at 12^+6^. (…) They told me that the previous doctor had made a mistake and that it was a very rare case (…). Then, at the *consultorio* [family planning centre] they told me: ‘Given that you’re 12^+6^, try anyway, because maybe we are still mistaken, go anyway [to the hospital] and try’.

At the local hospital, the head of OBGYN services confirmed that she had exceeded the legal GA limits and suggested she seek care in Spain or England. Carla ended up obtaining an abortion in England when she was 17 weeks pregnant.

## Discussion

### Main findings

Our study demonstrates that among people travelling for abortion from countries where this procedure is legal, the primary reason reported for going abroad was having exceeded gestational age limits in their country of residence. In our study population, 56% of participants travelling from such contexts reported having confirmed their pregnancy at 14 weeks or later; a point beyond the legal limit for abortion upon request in Italy, France, and Germany. A similar proportion of participants (58%) did not consider abortion as an option until after 14 weeks of pregnancy. Our qualitative data show that the participants who both confirmed their pregnancy and considered terminating it prior to the GA limits in their home copuntry were delayed by a number of barriers that eventually caused them to exceed these limits, including a lack of information, physicians’ unwillingness to provide abortion care and referral and, in some cases, gestational age miscalculation at the moment the pregnancy was confirmed.

### Strengths and limitations

This study fills a gap in the existing literature by providing new data on a topic that has been understudied despite its relevance from both a public health and a human rights perspective. It has been clearly documented that abortion restrictions and regulations can hinder access to safe abortion, increasing reproductive health risks by delaying care.^31^ Moreover, the need to travel in pursuit of healthcare services, given the cost, time and logistical concerns this entails, exacerbates existing inequities in healthcare access and resource distribution in healthcare systems and can have stratifying consequences for populations and individuals. In spite of this, no prior mixed‐methods study has documented or has explored the experiences of people travelling from countries with relatively liberal abortion laws to seek abortion care elsewhere within Europe.

Our study has limitations. First, our population is not a representative sample of all pregnant people who seek abortions in the countries where our study participants sought care. Moreover, it is not generalisable to the abortion experiences of all people who seek abortion in the countries where our study participants reside. Many pregnant people obtain abortions at below the GA limit in these countries, and some of those who are above the limit either self‐manage or carry to term. Additionally, we are not able to comment on the way that gestational age restrictions and other barriers may have impacted those who tried to seek care outside their country but who ultimately found the barriers to travel insurmountable, or those who sought underground procedures, self‐induced or attempted to do so, or who were forced to carry unwanted pregnancies to term. Finally, there are limits to our descriptive analytical approach. Further statistical analyses accompanied by in‐depth qualitative analyses of these data will give us a more nuanced understanding of the impacts associated with the need to travel for abortion at later gestational ages, and the barriers encountered when travelling. Finally, it is possible that our results may underestimate the proportion of participants whose primary motivation for travel was gestational age limits in their country of residence, as an additional 8% of participants indicated that abortion was ‘not legal in their country for their situation’, which could reflect an interpretation from some that gestational age limits were legal restrictions that applied to ‘their situation’.

### Interpretation

Results from our study align with findings from previously published research conducted with women who had travelled to England for an abortion from countries with relatively liberal abortion laws.^22^ That former study also found that gestational age limits were the main factor compelling women to travel for abortion care. Our study expands on those findings by providing insight into the experiences of women who travel to the Netherlands and Spain, in addition to those who travel to the UK, shedding light on the unique experiences and challenges faced by participants. We thereby highlight the need for an increased focus on policies that support access to abortion throughout pregnancy.

The continued existence of the need to travel abroad for abortion services among residents of countries with relatively liberal laws demonstrates that the legislation in countries such as Germany, Italy and France do not meet the needs of all people seeking abortion. Furthermore, it exposes the ways in which procedural, and regulatory restrictions meaningfully limit abortion access, even in countries where abortion is legal upon request or on broad grounds. Our findings suggest that existing restrictions on access to abortion beyond 12–14 weeks of gestation, as well as inconsistent interpretation and application of these laws, are one of the main forces constraining the ability of pregnant people legally to obtain abortion care in their country of residence.

## Conclusions

GA limits in countries where abortion is legal on broad grounds only until a certain point in the pregnancy appear to be antithetical to ensuring access for all people who need abortion care. The continued existence of cross‐border abortion‐related travel illustrates that early access to abortion is necessary but not sufficient to meet reproductive health needs of all people. GA limits restrict access to high‐quality abortion care in countries across Europe and can further delay pregnant people who encounter additional barriers in their search for pregnancy termination procedures, thus increasing their reproductive health risks.^31^ Additionally, our study documents the experiences of those who reach abortion clinics abroad but does not account for all those who may have tried to do the same but failed, who sought underground procedures, who self‐induced or attempted to do so or who were forced to carry unwanted pregnancies to term. Existing policies and authorities failed to ensure these women’s reproductive health needs were met. In the current context, the individual or collective circumstances which make travel difficult or even impossible (e.g. economic resources, migrant or visa status, global pandemic) further constrain people’s reproductive autonomy and negatively impact their ability to access abortion – an essential healthcare service. If GA limits were to be abolished, no pregnant person would need to travel across borders to another European country to find the care they need, resort to underground abortion options or carry an unwanted pregnancy to term simply because they found themselves seeking abortion services beyond the limit established by the legislation in their own country of residence.

### Disclosure of interests

None declared. Completed disclosure of interests forms are available to view online as supporting information.

### Contribution to authorship

SDZ is the PI on this research project. She designed the study, undertook data collection and analysis in Spain for the phase on cross‐border travel for abortion care, and supervised data collection and qualitative data analysis. She contributed to all drafts of the manuscript, and reviewed all authors’ contributions. GZ was a Post‐doctoral Fellow on this research project, responsible for all data collection and qualitative data analysis in the UK. She contributed to all drafts of the manuscript. JM is a Senior Researcher on this research project, and contributed to study design, supervision of data collection and qualitative data analysis. She contributed to all drafts of the manuscript, and reviewed all authors’ contributions. C Garnsey is a research assistant at Ibis Reproductive Health. She contributed to supervision of quantitative data collection in all sites, and quantitative data analysis, and contributed to all drafts of the manuscript. A‐KZ was a research assistant on this project responsible for data collection and qualitative data analysis in the Netherlands. She contributed to drafting the qualitative results section of this manuscript. C Gerdts is a Senior Researcher on this research project, and contributed to study design, supervision of quantitative data collection and quantitative data analysis. She contributed to all drafts of the manuscript, and reviewed all authors’ contributions.

### Details of ethics approval

Eight international scientific experts reviewed the research proposal and, after it was selected for funding, the study protocol was evaluated and approved by the ERC Ethics Committee on 4 March 2016: ERCEA/BT/ercea.b.1(2016)1090019. Ethical approval for this study was also granted by the University of Barcelona (Spain) on 13 February 2017, the University of Central Florida (US) on 21 February 2017 (SBE‐17‐12964), the University of Tilburg (Netherlands) on 23 March 2017 (EC‐2017.22) and the BPAS Research & Ethics Committee (UK) on 8 May 2017 (REC 2017/02/SDZ).

### Funding

This study is funded by the European Research Council (ERC) via a Starting Grant awarded to Dr De Zordo (BAR2LEGAB, 680004) and is hosted by the University of Barcelona. It is also supported by the Spanish Ministerio de Economía, Industria y Competitividad through grant RYC‐2015‐19206. The funders had no role in the design or conduct of the study; collection, management, analysis or interpretation of the data; preparation, review or approval of the manuscript; or the decision to submit the manuscript for publication.

### Acknowledgements

We would like to thank our study participants and the organisations and clinics that partnered with us in the design and development of the study, including the British Pregnancy Advisory Service (BPAS) in England, CASA Kliniek, Beahuis & Bloemenhove Kliniek and Rutgers in the Netherlands, Clínica d’Ara, Clínica Sants and Clínica Aragón in Catalunya (Spain). Finally, this study would have not been possible without the funds from the European Research Council and the support of the host institution, the University of Barcelona.

## Supporting information

Supplementary Material

Supplementary Material

Supplementary Material

Supplementary Material

Supplementary Material

Supplementary Material

## Data Availability

The data that support the findings of this study are available on request from the corresponding author. The data are not publicly available due to privacy or ethical restrictions.

## References

[1] The World’s Abortion Laws | Center for Reproductive Rights [Internet] [https://reproductiverights.org/worldabortionlaws]. Accessed 7 August 2019.

[2] Pinter B , Aubeny E , Bartfai G , Loeber O , Ozalp S , Webb A . Accessibility and availability of abortion in six European countries. Eur J Contracept Reprod Health Care 2005;10:51–8. 16036299 10.1080/13625180500035231

[3] Chavkin W , Swerdlow L , Fifield J . Regulation of conscientious objection to abortion: an international comparative multiple‐case study. Health Hum Rights 2017;19:55. 28630541 PMC5473038

[4] Levels M , Sluiter R , Need A . A review of abortion laws in Western‐European countries. A cross‐national comparison of legal developments between 1960 and 2010. Health Policy 2014;118:95–104. 25059743 10.1016/j.healthpol.2014.06.008

[5] Minerva F . Conscientious objection in Italy. J Med Ethics 2015;41:170–3. 24861043 10.1136/medethics-2013-101656

[6] De Zordo S , Mishtal J , Anton L . A Fragmented Landscape: Abortion Governance and Protest Logics in Europe (Protest, Culture & Society Book 20). New York: Berghahn Books; 2016.

[7] Lavelanet AF , Johnson BR , Ganatra B . Global abortion policies database: a descriptive analysis of the regulatory and policy environment related to abortion. Best Pract Res Clin Obstet Gynaecol 2020;62:25–35. 31300212 10.1016/j.bpobgyn.2019.06.002

[8] Ministerie van Volksgezondheid, Welzijn en Sport . Jaarrapportage Wet Afbreking Zwangerschap (Wafz) 2017 – Rapport – Inspectie Gezondheidszorg en Jeugd [Internet]. 2019 [https://www.rijksoverheid.nl/documenten/rapporten/2019/01/02/jaarrapportage‐wet‐afbreking‐zwangerschap‐2017]. Accessed 15 October 2019.

[9] Abortion statistics for England and Wales: 2018 – GOV.UK [Internet] [www.gov.uk/government/statistics/abortion‐statistics‐for‐england‐and‐wales‐2018]. Accessed 28 October 2019.

[10] Barr‐Walker J , Jayaweera RT , Ramirez AM , Gerdts C . Experiences of women who travel for abortion: a mixed methods systematic review. PLoS One 2019;14:e0209991. 30964860 10.1371/journal.pone.0209991 PMC6456165

[11] Francome C . Irish women who seek abortions in England. Fam Plann Perspect 1992;24:265–8. 1483530

[12] Rossiter A . Ireland’s Hidden Diaspora: The Abortion Trail and the Making of a London‐Irish Underground, 1980–2000, Pennsauken, NJ: Pennsauken BookBaby; 2009.

[13] Bloomer F , O’Dowd K . Restricted access to abortion in the Republic of Ireland and Northern Ireland: exploring abortion tourism and barriers to legal reform. Cult Health Sex 2014;16:366–80. 24617662 10.1080/13691058.2014.886724

[14] Loeber O , Wijsen C . Factors influencing the percentage of second trimester abortions in the Netherlands. Reprod Health Matters 2008;16(Suppl 31):30–6. 18772081 10.1016/S0968-8080(08)31377-9

[15] Ellertson C . Mandatory parental involvement in minors’ abortions: effects of the laws in Minnesota, Missouri, and Indiana. Am J Public Health 1997;87:1367–74. 9279279 10.2105/ajph.87.8.1367 PMC1381104

[16] Foster DG , Kimport K . Who seeks abortions at or after 20 weeks? Perspect Sex Reprod Health 2013;45:210–8. 24188634 10.1363/4521013

[17] Jones RK , Jerman J . How far did US women travel for abortion services in 2008? J Womens Health 2013;22:706–13. 10.1089/jwh.2013.4283 23863075

[18] Silva M , McNeill R . Geographical access to termination of pregnancy services in New Zealand. Aust N Z J Public Health 2008;32:519–21. 19076741 10.1111/j.1753-6405.2008.00302.x

[19] Forrest JD , Sullivan E , Tietze C . Abortion in the United States, 1977–1978. Fam Plann Perspect 1979;11:329–41. 401078

[20] Henshaw SK , O’Reilley K . Characteristics of abortion patients in the United States, 1979 and 1980. Fam Plann Perspect 1983;15(1):5–8, 10–6. 6394357

[21] Sanders JN , Conway H , Jacobson J , Torres L , Turok DK . The longest wait: examining the impact of Utah’s 72‐hour waiting period for abortion. Womens Health Issues 2016;26:483–7. 27502901 10.1016/j.whi.2016.06.004

[22] Gerdts C , DeZordo S , Mishtal J , Barr‐Walker J , Lohr PA . Experiences of women who travel to England for abortions: an exploratory pilot study. Eur J Contracept Reprod Health Care 2016;21:401–7. 27539129 10.1080/13625187.2016.1217325

[23] Gerdts C , Fuentes L , Grossman D , White K , Keefe‐Oates B , Baum SE , et al. Impact of clinic closures on women obtaining abortion services after implementation of a restrictive law in Texas. Am J Public Health 2016;106:857–64. 26985603 10.2105/AJPH.2016.303134 PMC4985084

[24] Jones RK , Upadhyay UD , Weitz TA . At what cost? Payment for abortion care by U.S. women. Womens Health Issues 2013;23:e173–8. 23660430 10.1016/j.whi.2013.03.001

[25] Jerman J , Frohwirth L , Kavanaugh ML , Blades N . Barriers to abortion care and their consequences for patients traveling for services: qualitative findings from two states. Perspect Sex Reprod Health 2017;49:95–102. 28394463 10.1363/psrh.12024 PMC5953191

[26] Baum SE , White K , Hopkins K , Potter JE , Grossman D . Women’s experience obtaining abortion care in Texas after implementation of restrictive abortion laws: a qualitative study. PLoS One 2016;11:e0165048. 27783708 10.1371/journal.pone.0165048 PMC5082726

[27] Sethna C , Davis G . Abortion Across Borders: Transnational Travel and Access to Abortion Services. Baltimore: JHU Press; 2019.

[28] StataCorp . Stata Statistical Software: Release 12. College Station, TX: StataCorp LP; 2011.

[29] Corbin JM , Strauss A . Grounded theory research: Procedures, canons, and evaluative criteria. Qual sociol. 1990;13:3–21.

[30] Autorino T , Mattioli F , Mencarini, L . The impact of gynecologists’ conscientious objection on abortion access. Soc Sci Res 2020;87:102403. 32279862 10.1016/j.ssresearch.2020.102403

[31] World Health Organization . Safe Abortion: Technical and Policy Guidance for Health Systems Evidence Summaries and Grade Tables. Geneva: World Health Organization; 2012.

